# Identification of potential anti-inflammatory components in Moutan Cortex by bio-affinity ultrafiltration coupled with ultra-performance liquid chromatography mass spectrometry

**DOI:** 10.3389/fphar.2024.1358640

**Published:** 2024-02-07

**Authors:** Caomin Zou, Qianru Chen, Jiasheng Li, Xiguang Lin, Xingyang Xue, Xinhang Cai, Yicheng Chen, Yue Sun, Shumei Wang, Ying Zhang, Jiang Meng

**Affiliations:** ^1^ School of Traditional Chinese Medicine, Guangdong Pharmaceutical University, Key Laboratory of Digital Quality Evaluation of Chinese Materia Medica, State Administration of Traditional Chinese Medicine (TCM), Engineering Technology Research Center for Chinese Materia Medica Quality of Universities in Guangdong Province, Guangdong Provincial Key Laboratory of Traditional Chinese Medicine Informatization, Guangzhou, China; ^2^ Affiliated Cancer Hospital and Institute of Guangzhou Medical University, Guangzhou, China; ^3^ College of Pharmacy, Jinan University, Guangzhou, China

**Keywords:** Moutan cortex, cox-2 inhibitors, bio-affinity ultrafiltration, UPLC-MS, anti-inflammatory

## Abstract

Moutan Cortex (MC) has been used in treating inflammation-associated diseases and conditions in China and other Southeast Asian countries. However, the active components of its anti-inflammatory effect are still unclear. The study aimed to screen and identify potential cyclooxygenase-2 (COX-2) inhibitors in MC extract. The effect of MC on COX-2 was determined *in vitro* by COX-2 inhibitory assays, followed by bio-affinity ultrafiltration in combination with ultra-performance liquid chromatography-mass spectrometry (BAUF-UPLC-MS). To verify the reliability of the constructed approach, celecoxib was applied as the positive control, in contrast to adenosine which served as the negative control in this study. The bioactivity of the MC components was validated *in vitro* by COX-2 inhibitor assay and RAW264.7 cells. Their *in vivo* anti-inflammatory activity was also evaluated using LPS-induced zebrafish inflammation models. Finally, molecular docking was hired to further explore the internal interactions between the components and COX-2 residues. The MC extract showed an evident COX-2-inhibitory effect in a concentration-dependent manner. A total of 11 potential COX-2 inhibitors were eventually identified in MC extract. The COX-2 inhibitory activity of five components, namely, gallic acid (GA), methyl gallate (MG), galloylpaeoniflorin (GP), 1,2,3,6-Tetra-O-galloyl-β-D-glucose (TGG), and 1,2,3,4,6-Penta-O-galloyl-β-D-glucopyranose (PGG), were validated through both *in vitro* assays and experiments using zebrafish models. Besides, the molecular docking analysis revealed that the potential inhibitors in MC could effectively inhibit COX-2 by interacting with specific residues, similar to the mechanism of action exhibited by celecoxib. In conclusion, BAUF-UPLC-MS combining the molecular docking is an efficient approach to discover enzyme inhibitors from traditional herbs and understand the mechanism of action.

## 1 Introduction

To date, an increasing body of evidence has suggested that chronic inflammation may be directly associated with the occurrence of cancer through its ability to induce tumorigenic transformation in susceptible cells (Federico et al., 2007). Typically, inflammatory process involves with injury and/or infection, as well as tissue regeneration and repair prior to inflamation resolution. Upon initiation of the inflammatory response, neutrophils and macrophages, which are inflammatory cells, undergo aggregation followed by the release of specific factors that facilitate the progression of inflammation (Fitzpatrick, 2001; Aggarwal et al., 2006; Bartsch and Nair, 2006). Cyclooxygenase-2 (COX-2), a crucial factor associated with inflammation that is less frequently expressed in normal organisms, has been observed to be induced by cytokines, mitogens, and endotoxins in inflammatory cells. It plays a pivotal role in the development of inflammation by increasing prostaglandin levels *in vivo* (Huai, et al., 2019). Moreover, epidemiological studies have underscored COX-2 as a pivotal area of interest in the development of cancer prevention strategies, and Nonsteroidal anti-inflammatory drugs (NSAIDs) have exhibited broad effectiveness by competitively suppressing the activity of cyclooxygenase enzymes. (Smalley and DuBois, 1997). While the long-term use of anti-inflammatory medications, particularly NSAIDs and corticosteroids, may lead to some side effects, ranging from cardiovascular issues to gastrointestinal discomfort and potential impacts on liver and kidney function (Bindu et al., 2020; Braun et al., 2020; Watanabe et al., 2020). Traditional Chinese Medicine (TCM) has been recognized for its anti-inflammatory efficacy with a relatively low incidence of side effects (Zhang et al., 2017). Consequently, it is important to systematically identify and validate the anti-inflammatory components from TCM with high-throughput and reliable methodologies.

Moutan Cortex (MC), the root bark of *Paeonia suffruticosa* Andr., is a traditional Chinese herb widely utilized in clinical settings for its analgesic, sedative, and anti-inflammatory properties. It is also used as a remedy for cardiovascular diseases, conditions with a manifestation of extravasated blood or stagnated blood, as well as female diseases such as menstruation disorders and uteritis (Yun et al., 2013). In-depth research on MC has unveiled its potential anti-inflammatory and antiviral properties (Sheehan and Atherton, 1994; Au et al., 2001; Hsiang et al., 2001). For example, Chun et al. discovered that the anti-inflammatory activity of MC could potentially be attributed to its inhibition of NO and PGE2 productional. Apart from that, it also led to a reduction in the concentration of TNF-α, IL-1β and IL-6 in LPS-activated cells by suppressing the expression of iNOS, COX-2, p-IκBɑ and NF-κB (Chun et al., 2007). However, despite the reports delineating the association between the anti-inflammatory activity and certain monomeric compounds in MC, there have been limited studies conducted to identify the active anti-inflammatory components with a COX-2-inhibitory effect. Nevertheless, it is undeniably of great significance to identify the COX-2-inhibiting compounds in MC and elucidate their underlying mechanism for further research and clinical applications.

The biologically active constituents derived from natural herbs possess potential applications in novel drug development or as lead compounds for structural modification and optimization (Mohan et al., 2021). The current methodology employed for the identification of active ingredients in natural plants primarily involves chemical separation and structure elucidation. However, this approach is often criticized for its lengthy cycle time and susceptibility to loss of active components, thus rendering it inherently flawed (Wolfender et al., 2015). So far, bio-affinity ultrafiltration coupled with ultra-performance liquid chromatography-mass spectrometry (BAUF-UPLC-MS) has been regarded as an efficient tool commonly used for rapid and efficient screening of active ingredients from complex natural product systems (Wei et al., 2016). Having a high affinity with the target molecules which can be traced by UPLC-MS, the active compounds are able to be separated from the solution system for further identification. For example, Lan successfully identified potential thrombin inhibitors from Curcuma Rhizoma using UPLC-Q-Exactive Orbitrap/MS and subsequently validated their thrombin-inhibiting efficacy through *in vivo* and *in vitro* assays (Lan et al., 2021).

**FIGURE 1:**
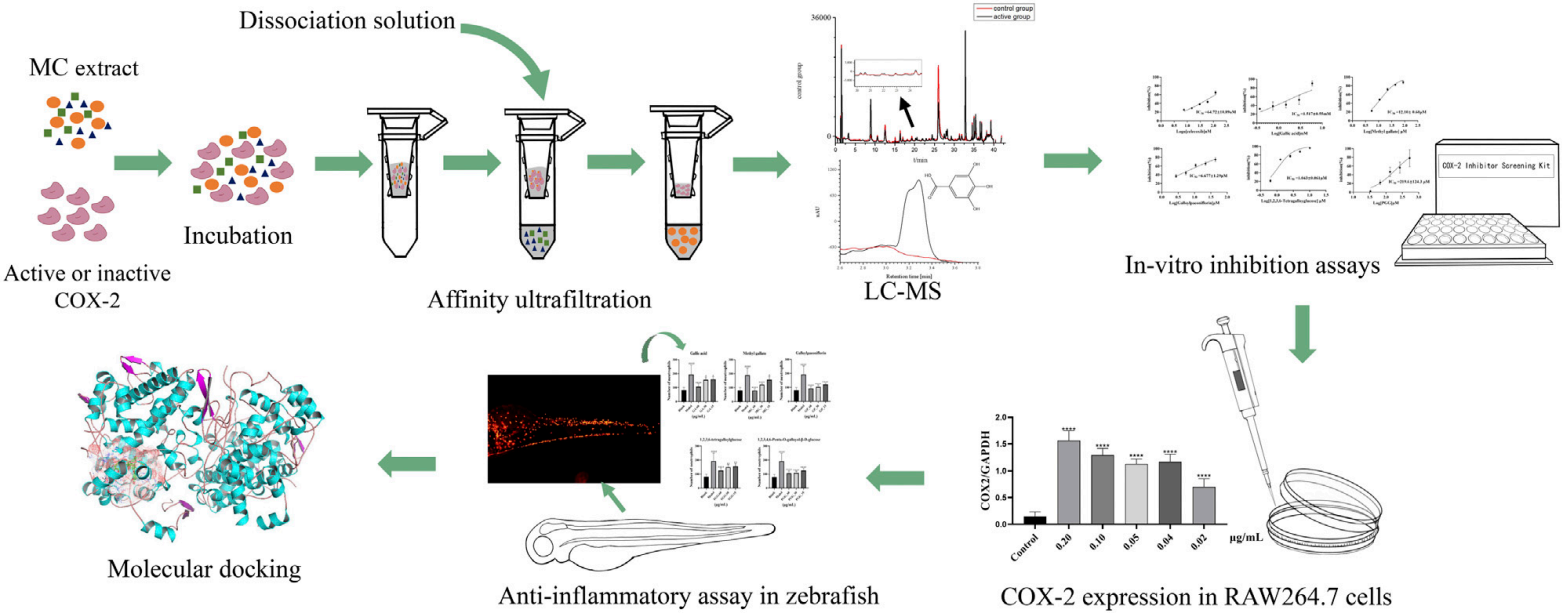
Schematic diagram of screening COX-2 inhibitors from MC by BAUF-UPLC-Q-Exactive Orbitrap-MS.

In this study, our aim was to identify the potential active anti-inflammatory compounds of MC and elucidate their underlying mechanisms. The inhibitory effect of MC on COX-2 was determined through COX-2-inhibitory assays, followed by BAUF-UPLC-MS analysis to identify potential components responsible for COX-2 inhibition in the MC extract. These components’ COX-2 inhibiting effects were further validated through additional COX-2 inhibitory assays and *in vivo* experiments using established zebrafish inflammation models to evaluate their anti-inflammatory efficacy. Finally, molecular docking techniques were employed to investigate the interaction between the identified components and COX-2. The procedure was illustrates in Figure 1.

## 2 Materials and methods

### 2.1 Materials and animals

Gallic acid (GA, batch number: CHB180114), 1,2,3,4,6-Penta-O-galloyl-β-D-glucopyranose (PGG, batch number: CHB190125), and 1,2,3,6-Tetra-O-galloyl-β-D-glucose (TGG, batch number: CHB201224) were purchased from Chengdu Chroma-Biotechnology Co., Ltd (Chengdu, China). Methyl gallate (MG, batch number: AF20061051) was obtained from Chengdu Alfa Biotechnology Co., Ltd (Chengdu, China). Galloylpaeoniflorin (GP, batch number: 220319) was from Chengdu Herb Substance Bio-Technology Co., Ltd (Chengdu, China). Celecoxib (batch number: D11A6S2151) was from Shanghai Yuanye Bio-Technology Co., Ltd (Shanghai, China). Adenosine (batch number: CSN23524-003) was purchased from CSNpharm, Inc (Chicago, United States). All the reagents were of HPLC grade.

MC (batch number: YPA7H0001) was obtained from Caizhilin Pharmaceutical Co., Ltd (Guangzhou, China) and identified by Professor Jizhu Liu from the School of Traditional Chinese Medical Materials, Guangdong Pharmaceutical University. The voucher specimens were preserved at the Herbarium Centre, Guangdong Pharmaceutical University. The samples, after transferred to a rocking pulverizer (DFy-400-D) and crushed into small pieces, were passed through an 80-mesh sieve and sealed for preservation. The herb powder was ultrasonically extracted once with 50% methanol (1:10, w/v), and then processed by a rotary evaporator as well as a freeze dryer to harvest the MC extract, which was sealed and then preserved under 4°C.

The COX-2 Inhibitor Screening Kits were procured from Beyotime Biotechnology (Shanghai, China). Human recombinant COX-2 (No.60122, 5000U) was obtained from Cayman Chemicals (Ann Arbor, MI, United States). Lipopolysaccharides (LPS, batch number: L118716) from *Escherichia coli* O55:B5 was provided by Shanghai Aladdin Biochemical Technology Co., Ltd. (Shanghai, China). Centrifugal ultrafiltration filters (Amico Ultra-0.5, 10 kDa) were from Millipore Co., Ltd. (Bed Ford, MA, United States).

Tg (lyz:DsRed2) zebrafish larvae from the National Zebrafish Resource Center were processed under lysozyme C (lyz) promoter to establish zebrafish models containing DsRed-labeled neutrophils (Xu et al., 2018). The models were employed in this experiment to assess the anti-inflammatory efficacy of the compounds based on the premise that zebrafish neutrophil count exhibited a positive correlation with proinflammatory substance concentration (Chen et al., 2019). The experiment was conducted in the major laboratory of digital quality evaluation of Chinese Materia Medica in Guangdong Pharmaceutical University (Guangzhou, China), where the zebrafish were kept in an automatic circulating tank system, with live brine shrimp as the feed twice a day. The conditions were maintained as a 14 h light/10 h dark cycle, with the water temperature controlled at 28°C ± 0.5°C and pH at 7.0 ± 0.5. After the zebrafish produced embryos naturally, the fertilized zebrafish eggs were gathered and processed with culture water. All procedures involving with Zebrafish experiments in this study were under the approval by the Institutional Ethics Committee (IEC) of Guangdong Pharmaceutical University (Ethics Approval number: GDPULAC2021121), and carried out according to the requirements of the International AAALAC certification (certification No. 001458).

### 2.2 Determination of COX-2 inhibitory activity

In this experiment, the inhibitory effect was determined by using COX-2 Inhibitor Screening Kits. A 96-well black plate was employed, where COX-2 cofactor working solution (5 μL), COX-2 assay buffer (75 μL), test solution (5 μL), and COX-2 working solution (5 uL) were mixed and incubated for 10 min at 37°C in the dark. Then 5 μL of probe solution, together with 5 μL of COX-2 substrate solution, was added immediately. After a 37°C incubation in the dark for another 5 min, the fluorescence value was determined at an excitation wavelength of 560 nm and emission wavelength of 590 nm. In this experiment, Celecoxib, a widely used COX-2 inhibitor, was employed in the positive control group for method validation, while 5 μL of dimethyl sulfoxide (DMSO), as the substitute of the test solution, was applied in the 100% enzyme activity control group. Besides, COX-2 Assay Buffer was used in the blank control group to replace COX-2 working solution. MC extract or potential inhibitors were dissolved with DMSO as the test solution in the sample groups. The inhibition rate of the samples was obtained by the formula shown as below:

$$\text{Inhibition activity }\left(\%\right)=\left(F_{1}\;–\;F_{2}\right)\;/\;\left(F_{1}\;–\;F_{3}\right)\times 100\%$$

Where, F_1_, F_2,_ and F_3_ respectively indicate the fluorescence value of the three groups—100% enzyme activity control group, sample group and blank control group.

### 2.3 Screening procedures of BAUF-UPLC-MS

The screening procedure was performed by referring to the previous report (Jiao et al., 2019). An experimental group and inactivated enzyme group were set up in this study for comparison. 40 μL of 100 mg/mL MC extract as well as 280 μL of COX-2 (100 U/mL) was transferred to EP tube in the experimental group, in contrast to the control group where 280 μL of inactivated COX-2 (boiled in the water bath for 30 min) was used as the substitute of the activated enzyme. The tubes from both groups were incubated at 37°C in the dark for 40 min, aiming to create an optimal condition under which the potentially suitable ligands would completely combined with the enzyme. A centrifugal filter was employed for ultrafiltration at 15000 × g for 15 min. Afterwards, 300 μL of PBS was added to the samples, and the unbound small molecules were removed by 15000 × g centrifugation for 15 min. To avoid interference caused by non-specific binding, the target-ligand compounds were shifted to new ultrafiltration membranes, where 300 μL of methanol was added to the complexes and the compounds were subsequently maintained at room temperature for 15 min to allow dissociation of the ligands from COX-2. The procedure was repeated for three times. The samples, under a gentle stream of nitrogen, were evaporated to dryness at room temperature and redissolved in methanol for UPLC-MS analysis.

UPLC-Q-Exactive-Orbitrap-MS was performed to analyze the compounds in the filtrate using Thermo Scientific Orbitrap Fusion Tribrid Mass Spectrometer (Thermo Fisher Scientific, United States) and Ultimate 3,000 Ultra-performance liquid chromatograph, at the same time with Waters ACQUITY UPLC BEH RP18 (2.1 mm × 100 mm, 1.7 μm) employed for chromatographic analysis. The UPLC was conducted under such conditions: Gradient elution was carried out at a flow rate of 0.2 mL/min with water (containing 0.1% formic acid; A) and acetonitrile (B) as the mobile phases. The gradient program was set as 0–10 min, 5%–8% B; 10–12 min, 8%–12% B; 12–15 min, 12%–14% B; 15–20 min, 14%–16% B; 20–25 min, 16%–18% B; 25–30 min, 18%–22% B; 30–35 min, 22%–40% B; 35–38 min, 40%–95% B; 38–40 min, 95%–5% B; 40–42 min, 5% B. The column temperature was adjusted to 35°C and the injection volume of the samples was determined as 2 μL. The measurements were read at the detection wavelength of 254 nm. In the MS test, electrospray ion (ESI) sources were hired to analyze the compounds, with Full MS/dd MS^2^ used as the scanning mode; The other parameters of MS analysis were as follows: nebulizer voltage, 4.0 kV; sheath gas pressure, 35 arb; aux gas pressure, 15 arb; ion transport temperature, 320°C; evaporation temperature, 320°C; and CES, 10 eV. The average EPI scanning spectrum was observed at a CE of 15, 35 and 45, respectively. In addition, mass spectrometry as well as data acquisition was conducted by Orbitrap Fusion Tune and Xcalibur 4.0. By comparing peak areas of two groups, specific ligands binding to COX-2 were determined. Relative binding degree (BD) of COX-2 ligands were calculated by the following formula: BD (%) = (A1–A2)/A1×100%, Where A1and A2 were the peak area of compounds in the active enzyme group and the control denatured enzyme group, respectively (Qin et al., 2015; Cai et al., 2020).

### 2.4 Method validation

To evaluate the specificity of the established approach, a solution of celecoxib (specific COX-2 inhibitor) was prepared as the positive control, in contrast to adenosine which was used as the negative control, both at a concentration of 20 μM. The control samples were prepared with inactivated COX-2. Besides, comparison of the peak area of the ultrafiltrate was performed between the active and inactivated enzyme groups to determine the specific binding between ligands and COX-2.

HPLC condition: HPLC (Shimadzu Corporation, Japan) furnished with a binary pump, vacuum degasser, diode array detector (DAD), and automatic sampler, was employed in combination with the usage of an Ultimatetm XB-C18 (250 × 4.6 mm, 5 μm) to verify the affinity ultrafiltration approach. With reference to the modified approach recorded in the previous study (Jadhav and Shingare, 2005), the conditions for HPLC were set as follows: mobile phases, water (containing 0.1% formic acid; A) and acetonitrile (B); elution program, 0–30 min, 5%–95% B; column temperature, 35°C; sample injection volume, 20 μL; detection wavelength, 254 nm; and the flow rate, 1.0 mL/min.

### 2.5 COX-2 expression assay

#### 2.5.1 Cell culture

RAW264.7 cells originated from mouse macrophage cell line were purchased from the American Type Culture Collection. DMEM medium containing 5% fetal bovine serum and 1% Penicillin G Streptomycin was used to culture the cells under 37°C in a humidified atmosphere of 5% CO_2_.

#### 2.5.2 Cell viability assay (MTT)

Cell viability was determined by MTT assay, where RAW264.7 cells (6 × 10^5^ cells/well), after mixed with LPS or potential COX-2 inhibitors, were cultured in a 96-well plate for 12 h. Then DEME with 20% MTT solution was added, followed by a 37°C incubation for extra 4 h. After the supernatant was removed, DMSO was added to dissolve the insoluble formazan product. The optical density was measured by a microplate reader at 490 nm. In this experiment, the formula used to calculate cell viability was as follows:

$$\text{Cell viability }\left(\%\right)=\left({\text{OD}}_{\text{experimental}\;\text{group}}–\;{\text{OD}}_{\text{blank}\;\text{group}}\right)/\left({\text{OD}}_{\text{control}\;\text{group}}–\;{\text{OD}}_{\text{blank}\;\text{group}}\right)\times 100\%$$

#### 2.5.3 RT-qPCR test

RAW264.7 cells were seeded in a 6-well plate and cultured until they sticked to the wall. After a 12-h pretreatment with 0.2 μg/mL LPS, the cells were further treated with the potential inhibitors at a series of concentrations for 12 h till they were lysed. Then AG RNAex Pro Reagent was used to isolate the total RNA. A reverse transcription kit (Evo M M-LV RT Mix with gDNA Clean for qPCR, Accurate Biology China) was employed to convert RNA to cDNA. SYBR Green Premix Pro Taq HS qPCR kit (Accurate Biology, China) was applied to carry out q-PCR where the relative amount of mRNA was calculated with 2^-△△t^ method. The primer sequences were as follows:

GAPDH:

5′-ACT​CCA​CTC​ACG​GCA​AAT​TCA​AC-3′ (Forward)

5′-ACA​CCA​GTA​GAC​TCC​ACG​ACA​TAC-3′ (Reverse)

COX-2:

5′-CAC​ATT​TGA​TTG​ACA​GCC​CAC​CAA​C-3′ (Forward)

5′-AGT​CAT​CAG​CCA​CAG​GAG​GAA​GG-3′ (Reverse)

#### 2.5.4 Western blot analysis

RAW264.7 cells were resuspended over ice in 98% ice-cold radioimmunoprecipitation assay (RIPA) lysis buffer (Meilunbio, Shanghai, China) supplemented with 1% EDTA-free protease and 1% PMSF for 30 min, followed by a 25-min centrifugation at 12000 r/min, 4°C. Then the supernatant was removed, and bicchoninic acid (BCA) protein assay kit (Biosharp, Shanghai, China) was employed to measure the concentration of proteins. Afterwards, 5 × SDS-PAGE loading buffer (Biosharp, Shanghai, China) was added into the mixture, which was subsequently boiled to denature the proteins. Meanwhile, 7.5% SDS-polyacrylamide gel electrophoresis (SDS-PAGE) was used to separate equal amount of proteins for electroblotting on a 0.2 μM PVDF membrane (Millipore). The samples were then blocked with 5% BCA blocking buffer for 1 h, followed by an overnight 4°C incubation with the primary antibodies against COX-2 (RT1159, Huabio) and GAPDH (60004-1-lg, Proteintech). Thereafter, TBST was used to wash the membranes for four times, and HRP-labelled goat anti-mouse IgG (A2016, Beyotime, China) secondary antibody was transferred to the membranes for another 37°C incubation for 40 min. The expression was determined by enhanced chemiluminescent (ECL) reagent (MA0186-1, Meilunbio) in Gel Analyzer—a fully automated chemiluminescence image analysis system, and the intensity of the protein bands was evaluated by densitometry and quantified using ImageJ software with GAPDH as an internal reference.

#### 2.5.5 Statistical analysis

In the study, the data were mostly presented as mean ± SD. GraphPad Prism software was employed for statistical analysis, with one-way ANOVA applied for inter-group comparison among multiple groups, and *t*-test for comparison between pair groups.

### 2.6 Anti-inflammatory assay in zebrafish

With an attempt to further verify whether the compounds bear an *in vivo* anti-inflammatory effect, LPS was used to construct zebrafish inflammatory models for anti-inflammatory test. According to the method of model construction proposed in previous study (Li et al., 2018), healthy transgenic Tg (lyz: DsRed2) zebrafish larvae were randomly collected and transferred into a 12-well plate (n = 20/well) at 72 hpf. The samples were treated with the following protocols: (1) The control group was treated with 4 mL of 0.1% DMSO for 24 h; (2) The model group, 4 mL of 0.025 mg/mL LPS (dissolved in 0.1% DMSO); (3) The drug groups, in the wake of a treatment with 0.025 mg/mL LPS for 30 min, were supplemented with MC to a working concentration of 15, 30 and 60 μg/mL, respectively, and maintained for 24 h. The concentration gradient was designed based on the maximum tolerable concentration. Afterwards, approximately 15 zebrafish were respectively transferred from each of the groups to fresh water for observation and imaging using a fluorescence microscope (Olympus, Tokyo, Japan). In addition, Image-Pro Plus was also used to record the number of neutrophils.

### 2.7 Molecular docking

To obtain a three-dimensional view visualizing the interaction between COX-2 and the potential inhibitors, molecular docking technology was adopted to perform a computer simulation. The crystal structure of COX-2 (code ID:1CVU, resolution:2.40 Å) was obtained from the RCSB Protein Data Bank in PDB format (Kiefer et al., 2000). By submitting the PDB file to the “build/check/repair model” and “Prepare PDB file for docking programs” modules, some repair work was further completed, such as modeling the missing side chains, performing a small regularization, correcting water positions and symmetry, and adding hydrogen. The fixed PDB file was then assessed and submitted to AutodockTools (ADT ver.1.5.6) to prepare PDBQT file, and Gasteiger charges were computed for protein atoms. Then, the central coordination of the docking box was positioned at x = 24.952; y = 23.300; z = 48.666, and the size of the box was set at 60 × 60×60, 0.375 Å spacing. Created by ChemDraw, the ligand compounds from MC extract were then converted to mol2 format by Chem3D. Docking simulations were performed by ADT. The searching work, with lamarckian genetic algorithm (GALS) as the engine, was carried out in 100 runs. The docking procedure was run by Autodock4 using the region of the grid box, which was also employed by Autogrid4 to prepare affinity grid maps. The most stable docking model, which was identified for each of the components based on the beat-scored conformation under the ADT prediction, was applied to further analyze the binding mode of the molecular docking. PyMOL was used for analysis of the docking sites so that the interaction between enzymes and inhibitors could be observed and evaluated.

## 3 Result and discussion

### 3.1 Determination of the COX-2-inhibitory activity of MC extract

**FIGURE 2:**
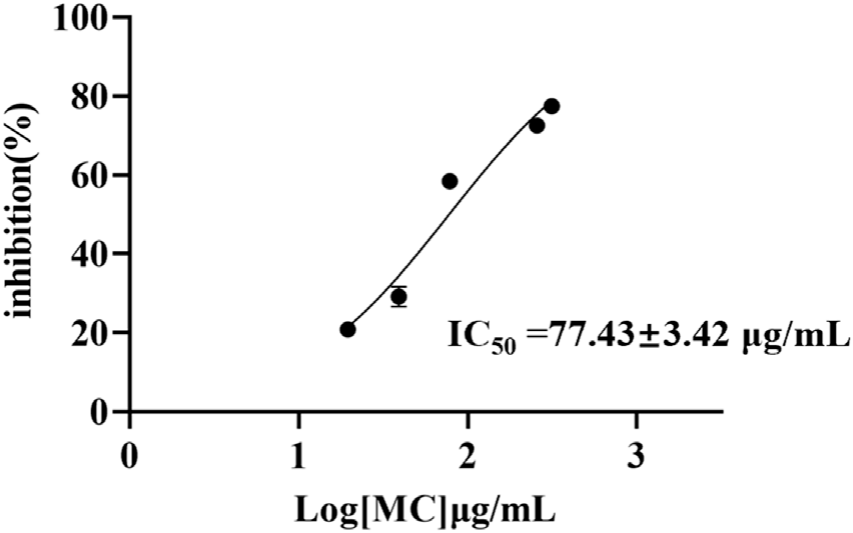
Dose-response curve of *in vitro* inhibition assay for Moutan Cortex extract.

To characterize the *in vitro* inhibitory effect of MC on COX-2, the inhibitory rate of MC extract was assessed at various concentrations using COX-2 Inhibitor Screening Kits. The resulting IC_50_ curve was generated using GraphPad Prism 9 software. The IC_50_ of MC extract is 77.43 ± 3.12 μg/mL. As depicted in Figure 2, an increase in the concentration of MC extract corresponded to a proportional elevation in its COX-2 inhibition rate, suggesting that the MC extract likely contains concentration-dependent potential COX-2 inhibitors.

### 3.2 Validation of affinity ultrafiltration method

**FIGURE 3:**
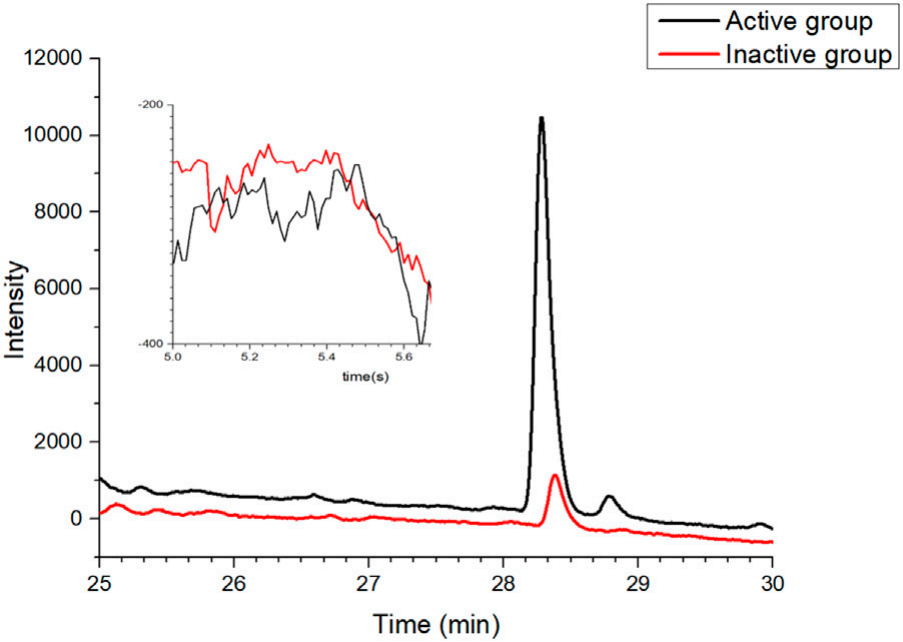
Validation of the affinity ultrafiltration in which mixtures of positive and negative drugs were tested to verify the feasibility of the established method: red line, control group with the inactivated enzyme; black line, experimental group with the active enzyme.

Celecoxib, a selective COX-2 inhibitor commonly used in clinical practice, was applied as the positive control in this study, while adenosine was used as the negative control. The two agents were mixed and subjected to affinity ultrafiltration aiming to verify the feasibility of the established approach. As shown in Figure 3, Celecoxib and adenosine were detected at 28.2 min and 5.5 min, respectively. Compared with the control group, the response value of celecoxib significantly increased in the active group. By contrast, there was no evident change in the response value of adenosine, suggesting that the established affinity ultrafiltration approach harbored a satisfying specificity for COX-2 test.

### 3.3 Screening potential COX-2 inhibitors by BAUF-UPLC-MS

The potential COX-2 inhibitors preliminarily identified in the experiment for determining COX-2-inhibitory activity of MC extract were further screened by BAUF-UPLC-MS. Following affinity ultrafiltration, the chromatographic peak areas were compared between the active-enzyme group and inactive-enzyme group, in an attempt to identify the bioactive components in the extract. Moreover, the reliability of the established approach was verified by setting up positive and negative control groups, which were also used to eliminate the possible disturbance produced by other inactive compounds.

**FIGURE 4:**
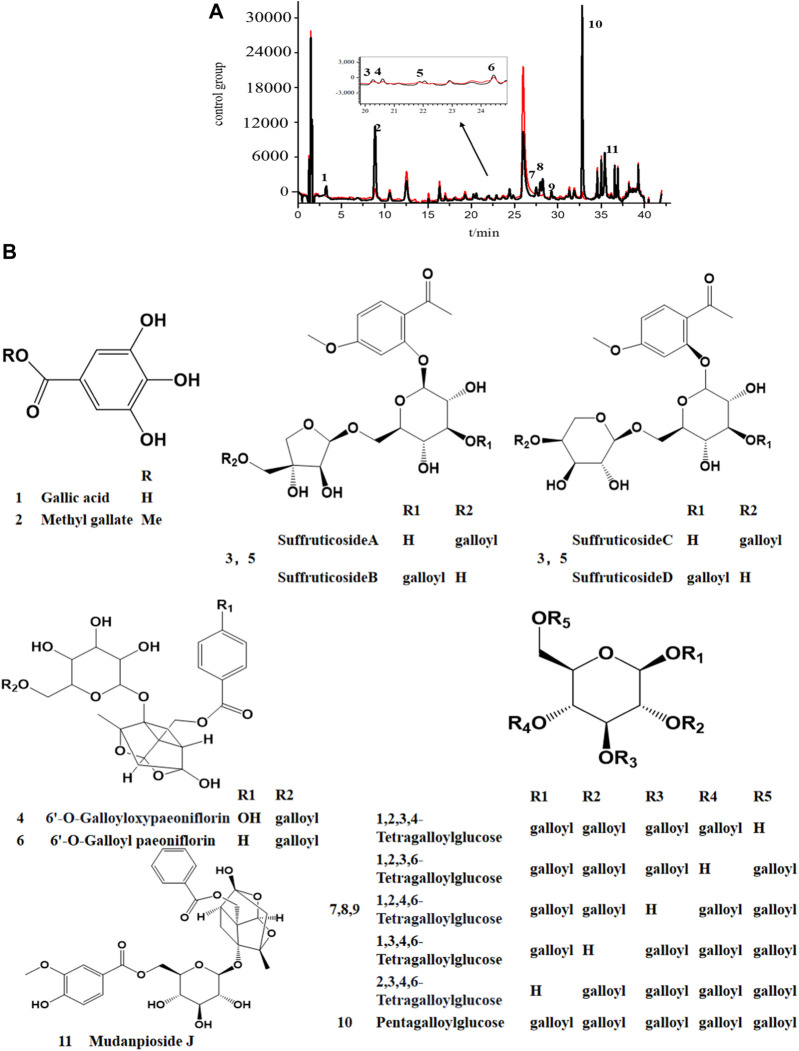
UPLC chromatogram **(A)** and the stuctures of potential COX-2 inhibitors **(B)** in MC by AUC. black line, the experimental group with active enzyme; red line, control group with denatured enzyme. **1**. Galic acide; **2**. Methy gallate; **3**. Suffruticoside A/B/C/D; **4**. 6′-O-Galloyloxypaeoniflorin; **5**. Suffruticoside A/B/C/D; **6**. 6′-O-Galloyl paeoniflorin; **7**. TGG; **8.** TGG; **9**. TGG; **10**. PGG; **11**. Mudanpioside J.

The UPLC chromatogram showed distinct differences in the two groups of the activated enzyme group (Figure 4A), and components in the activated group exhibited bigger peak areas than those in the inactivated enzyme group, which suggested possible COX-2 ligands in MC. The binding degree (BD) is defined to rank the affinity binding between the ligands and the enzyme. A higher BD value indicates stronger binding with the receptor and consequently greater inhibitory effect on the enzyme activity. Through analyzing the peak areas of two groups, the BDs for these identified compounds were calculated (Table 1), and the results showed that the BDs of 11 components more than 30%, which suggested that these compounds might be effective COX-2 ligands. However, it was also observed that some of the peak areas in the inactivated enzyme group were larger than those of the active enzyme group, which could possibly attribute to the fact that the protein spatial structure (about 74 kDa) was modified during inactivation of the enzyme in the boiling water bath. Therefore, when the inactivated enzyme was incubated with the extract, the pores in the ultrafiltration membrane were blocked and certain small unbound molecules were retained on the ultrafiltration membrane during the elution process (Zhang et al., 2021).

**TABLE 1:** Identification of potential COX-2 inhibitors in Moutan Cortex by UHPLC-Q-Exactive Orbitrap/MS.

No.	t_R_ (min)	Molecular formula	m/z	Measured ions	Mass error/ppm	Fragment ions	Tentative identification
1	3.44	C_7_H_6_O_5_	169.0134	[C_7_H_5_O_5_]^-^	1.362	125.0232	Gallic acid
2	8.99	C_8_H_8_O_5_	183.0291	[C_8_H_7_O_5_]^-^	1.367	168.0056, 124.0153	Methyl gallate
3	20.25	C_27_H_32_O_16_	611.1618	[C_27_H_31_O_16_]^-^	1.978	445.0986, 343.0666, 169.0133	Suffruticoside A/B/C/D
4	20.71	C_30_H_32_O_16_	647.1620	[C_30_H_31_O_15_]^-^	2.146	399.0931, 313.0564, 271.0459, 169.0133	6-O-Galloyloxy-paeoniflorin
5	21.97	C_27_H_32_O_16_	611.1618	[C_27_H_31_O_16_]^-^	1.863	445.0986, 301.0563, 169.0132, 165.0547	Suffruticoside A/B/C/D
6	24.94	C_30_H_32_O_15_	631.1668	[C_30_H_31_O_15_]^-^	1.685	613.1564, 313.0565, 177.0547, 169.0132	Galloylpaeoniflorin
7	27.40	C_34_H_28_O_22_	787.1001	[C_34_H_27_O_22_]^-^	1.666	635.0888, 617.0781, 483.0779, 465.0672, 447.0570, 169.0132	Tetragalloyl glucose
8	28.05	C_34_H_28_O_22_	787.1000	[C_34_H_27_O_22_]^-^	1.514	635.0888, 617.0784, 483.0781, 465.0671, 447.0566, 169.0132	Tetragalloyl glucose
9	28.33	C_34_H_28_O_22_	787.0998	[C_34_H_27_O_22_]^-^	1.272	635.0891, 617.0782, 483.0777, 65.0675, 447.0571, 169.0133	Tetragalloyl glucose
10	32.88	C_41_H_32_O_26_	939.1109	[C_41_H_31_O_26_]^-^	1.164	769.0892, 617.0784, 635.0894, 169.0133	1,2,3,4,6-Penta-O-galloyl-β-D-glucose
11	35.42	C_31_H_34_O_14_	629.1874	[C_31_H_33_O_14_]^-^	1.523	477.1401, 431.1346, 281.0665, 137.0232	Muddanpioside J

### 3.4 Identification of the potential COX-2 inhibitors by UPLC-Q-TOF-MS

**FIGURE 5:**
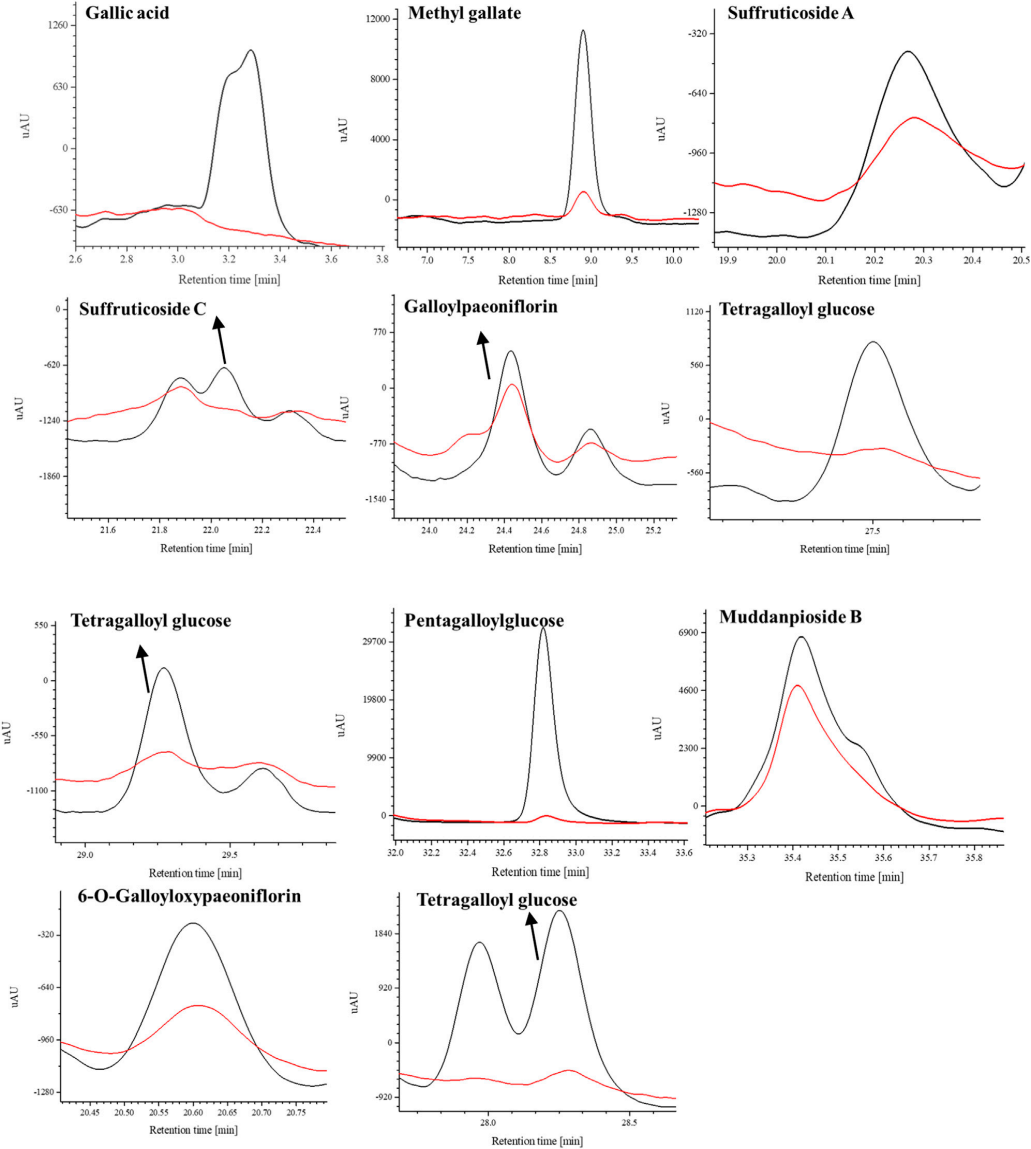
The selective ion monitoring (SIR) chromatograms (blue line, experimental group with active COX-2 enzyme; red line, control group with inactive COX-2 enzyme).

By referring to standard compounds and relevant findings from previous literature (He et al., 2006; He et al., 2010), the components of the BAUF ultrafiltrate were identified and characterized in this study based on their precise mass and fragment ions. The structure and the major data of the compounds, which were identified as monoterpene glycosides, galloyl glucoses, gallic acid, and their derivatives, were shown in Figure 4B; Figure 5; Table 1, respectively. Compund 1 and 2 wer derivatives of agllic acid; Compound 3, 4, 5, 6 and 11 were monoterpene glycosides; compund 7, 8, 9 and 10 are galloyl glucoses.

The mass spectrometry analysis revealed the loss of a higher number of water molecules, as well as small CO2 and CO molecules. Additionally, gallic acid and galloyl fragments were found to be easily detached from these compounds, which can be attributed to the presence of hydroxyl and carboxyl groups in the structure of gallic acid (Fernandes et al., 2022; Liu et al., 2023).

**FIGURE 6:**
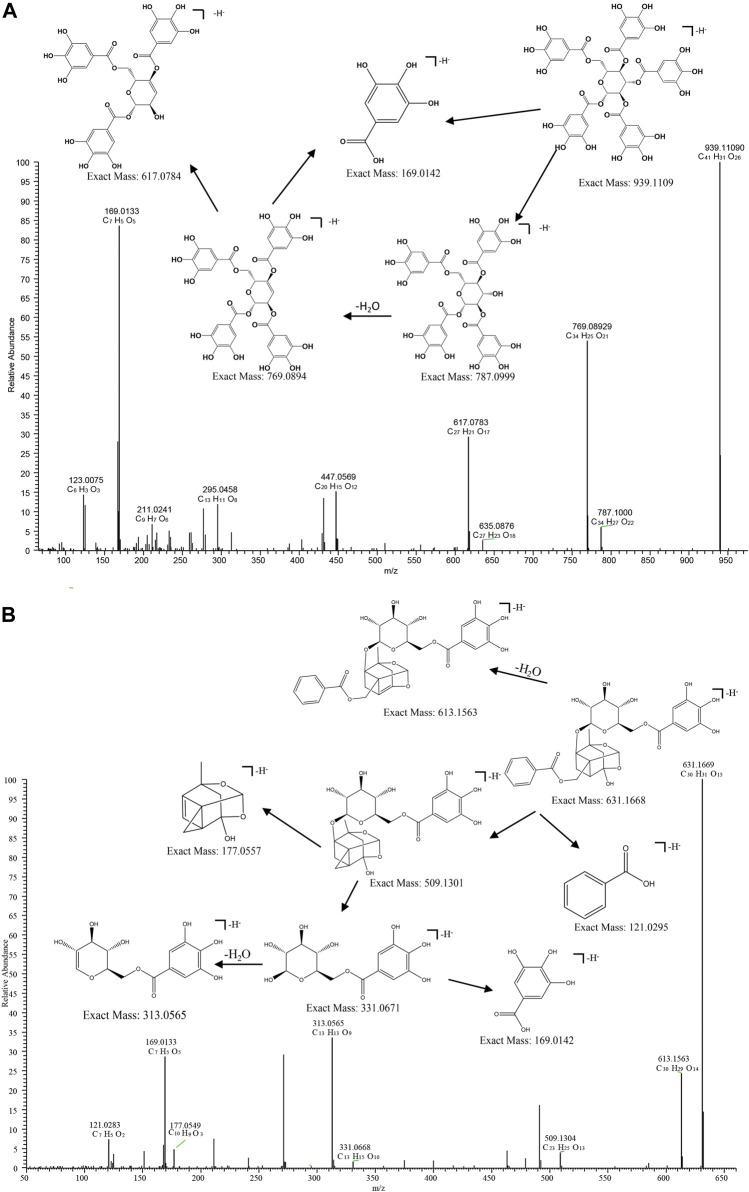
MS/MS spectrum and fragmentation pattern of PGG **(A)** and Galloylpaeoniflorin **(B)** from MC in negative ion mode.

Taking compound 10 as an example, it yielded a quasi-molecular ion peak of m/z 939.1109 [M-H]^-^ according to its MS/MS spectrum, and its molecular formula was identified as [C_41_H_31_O_26_]^-^ (1.164 ppm) by Xcalilbur. Successive loss of the fragment ions of galloyl (m/z 151.0027 [C_7_H_3_O_4_]^-^ (0.761 ppm)) and gallic acid (m/z 169.0133 [C_7_H_5_O_5_]^-^ (0.899 ppm)) were observed, in addition to subsequently identified fragments m/z 787.10040 ([M-galloyl]^-^) (1.971 ppm) and m/z 769.08936 ([M-gallic acid]^-^) (1.399 ppm). Therefore, the structure of compound 10 could be deduced as PGG (1,2,3,4,6-Penta-O-galloyl -β-D-glucopyranose)[27]. The proposed fragmentation pathway of PGG was shown in Figure 6.

The acetal-caged pinane skeleton is known as the characteristic construction in monoterpene glycosides. Various pinane backbone ion fragments can be seen in paeoniflorin compounds. The bonds attaching to glucosyl moiety, the ester group attaching to pinane backbone, as well as the ketal, methoxyl and carbonyl groups on aglycone are the sites where cleavage typically occurs, thereby, the neutral fragments of benzoic acid, glucose, CO, H_2_O, and formaldehyde are easily lost. compound 6 serves as a representative example, compound 6 yielded a quasi-molecular ion peak of m/z 631.1668 [M-H]^-^ ([C_30_H_31_O_15_]^-^). The fragment ions at m/z 613.1565 ([C_30_H_29_O_14_]^-^), 331.0671 ([C_13_H_13_O_9_]^-^) and 121.0295 ([C_7_H_6_O_2_]^-^) were indicative of the successive loss of H2O, galloyl glucose, and a benzoyl group, respectively. Then the fragment at m/z 331.0671 gave rise to 169.0134 ([M-C_6_H_12_O_5_]^-^) by successive loss of a glucose reside. by comparing its MS/MS behaviors with the reference standard and literature, the composition 6 was identified as galloylpaeoniflorin (Xiong et al., 2021). The proposed fragmentation pathway of galloylpaeoniflorin was shown in Figure 6.

Some studies have indicated that galloylate derivatives have antifungal property and also act as an antioxidant *in vivo* (Strippoli et al., 2000; Ow and Stupans, 2003). PGG has proved to have a potential to relieve pain and accelerate the recovery of voluntary movement that was constrained due to inflammatory pain, which was achieved by inhibiting generation of cellular NO in RAW 264.7 cells (Chun et al., 2016). Monoterpene glycosides, the main class of compounds in MC, have been recognized as a major contribution to the therapeutic effects of MC (Ding et al., 2012a; Ding et al., 2012b). Galloylpaeoniflorin attenuated osteoclast genesis and alleviates ovariectomy-induced osteoporosis by supressing ROS and MAPKs/c-Fos/NFATc1 signaling pathways (Liu et al., 2021). Plus, suffruticoside A/C have shown the potence associated with radical scavenging (Matsuda et al., 2001). These findings suggest that these potential COX-2 inhibitors could be used as leading compounds for a variety of biologically active products.

### 3.5 *In Vitro* COX-2 inhibition assays

**FIGURE 7:**
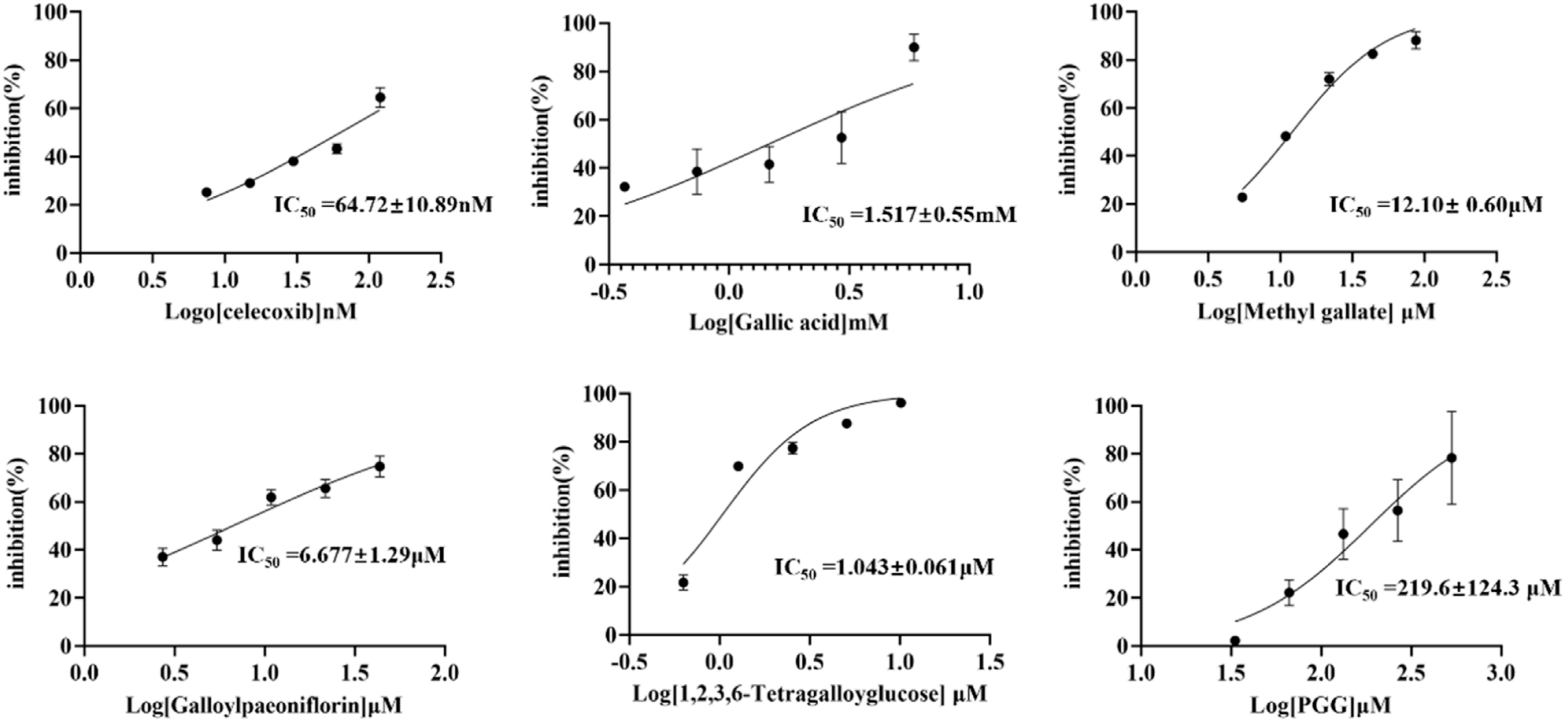
Dose-response curves of *in vitro* inhibition assays for celecoxib and five COX-2-inhibitory compounds.

To validate the experimental results of affinity ultrafiltration, the compounds identified in this study-gallic acid, methyl gallate, TGG, galloylpaeoniflorin, and PGG, as well as the positive control celecoxib, were also purchased from commercial marketsand. The COX-2 inhibiting activity of thees components were determined by COX-2 inhibitor screening kits. The dose-response curves as well as the half-maximal inhibitory concentrations (IC_50_ values) of each compound were shown in Figure 7. With Celecoxib as the positive control (IC_50_ = 0.0647 μM), TGG exhibited the most potent inhibitory effect on COX-2 activity (IC_50_ = 1.043 μM) among the five compounds screened in the affinity ultrafiltration study. Meanwhile, galloylpaeoniflorin (IC_50_ = 6.77 μM) and Methyl gallate (IC50 = 12.10 μM) demonstrated stronger inhibitory activities, while PGG and gallic acid displayed weaker inhibitory effects with IC_50_ values of 219.6 μM and 1.5170 mM, respectively. Unfortunately, due to the low content in this plant and difficulties in the process of enrichment and purification, there was not enough Suffruticoside A/B/C/D, Galloyloxypaeoniflorin and Muddanpioside J in our laboratory for the bioassay. The COX-2 inhibitory results suggest that BAUF-UPLC-MS, as an approach for identifying potential COX-2 inhibitors from MC extract, is capable and effective.

### 3.6 Anti-inflammatory assay in cells

**FIGURE 8:**
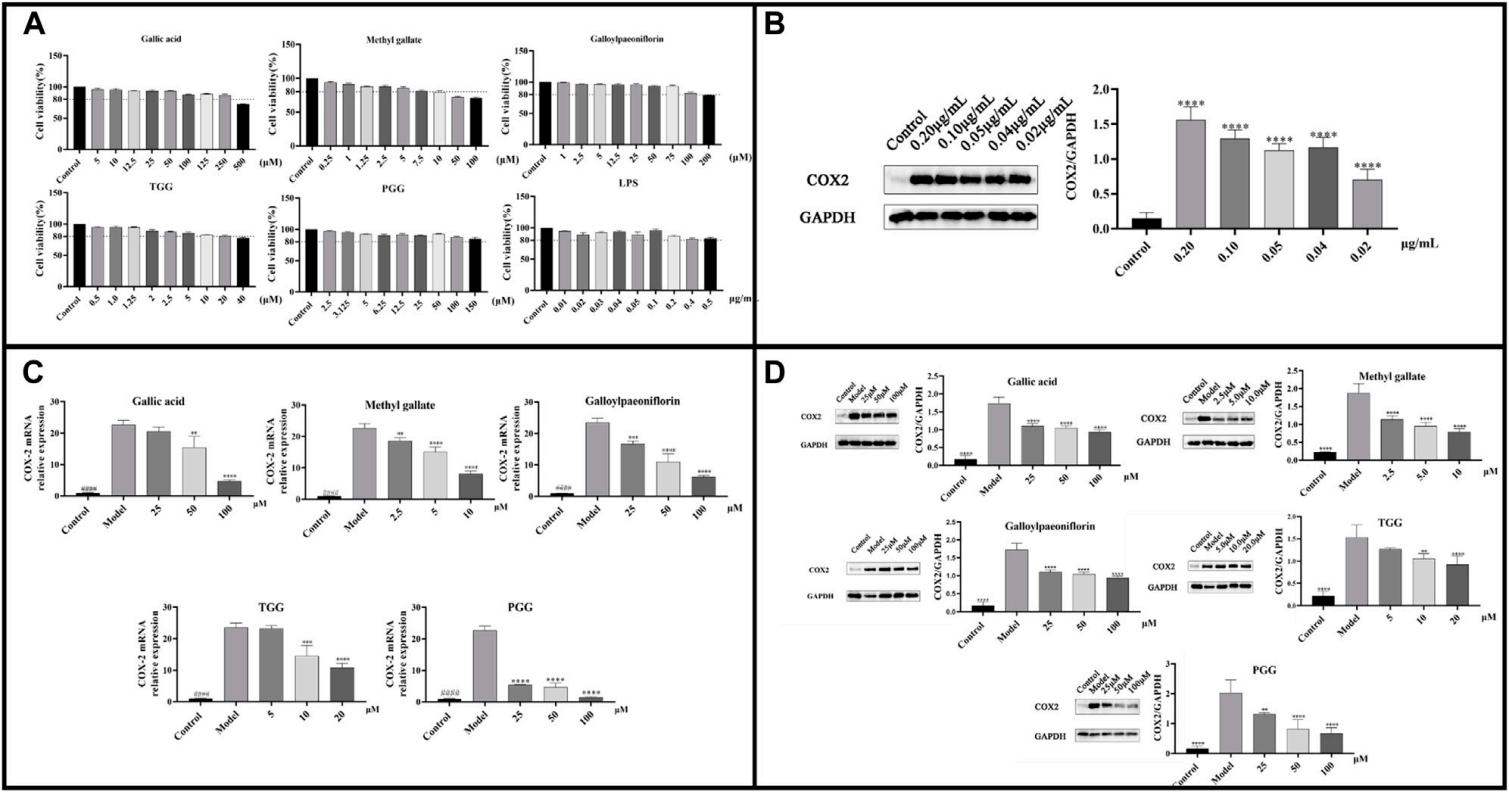
**(A)** Cell viability of LPS and each potential inhibitors by MTT assay; **(B)** COX-2 protein expression at different concentrations of LPS-induced RAW264.7; **(C)** COX-2 mRNA expressions in LPS-stimulated RAW264.7 cells following treatment by the potential inhibitors; **(D)** COX-2 Protein expressions in LPS-stimulated RAW264.7 cells following treatment by the potential inhibitors. ^####^ *p* < 0.0001 model group vs. blank group; ****p < 0.0001, ***p < 0.001, **p < 0.01 experimental group vs. model group.

The cell-based study aimed to elucidate the anti-inflammatory effect of the potential inhibitors in MC extract by exploring their activity in modulating COX-2 mRNA and protein expression using LPS-induced RAW264.7 cells. The anti-inflammatory activity was evaluated following the MTT assay, which was utilized to determine the cytotoxic concentration of LPS and potential inhibitors. Figure 8A demonstrated that Gallic acid (≤250 μM), Methyl gallate (≤10 μM), Galloylpaeoniflorin (≤100 μM), TGG (≤20 μM), PGG (≤150 μM), and LPS (≤0.5 μg/mL) exhibited no cytotoxicity against RAW264.7 cells. To identify the appropriate concentration for modeling, COX-2 expression in RAW264.7 cells was assessed by western blotting using LPS at a series of concentrations. Figure 8B showed that COX-2 protein expression was upregulated as the LPS concentration elevated, with the highest protein expression observed at 0.2 μg/mL. Subsequently, the effects of potential inhibitors on COX-2 mRNA and protein expressions were evaluated in LPS-treated RAW264.7 cells by RT-qPCR and western blotting technique. Figure 8C revealed that according to RT-qPCR, the COX-2 mRNA expression of the model group was evidently higher than that of the blank group. However, the COX-2 mRNA expression in groups treated with the potential inhibitors was lower as compared to that in the model group, indicating that these inhibitors showed a dose-dependent suppression on the expression of COX-2 gene. Figure 8D illustrated that COX-2 protein expression was dramatically higher in the model group compared with the blank group. Moreover, the COX-2 protein expression of the potential inhibitors groups was lower in comparison to that of the model group, which was consistent with the findings from RT-qPCR. These results suggested that the potential inhibitors were likely to exert anti-inflammatory effect by modulating LPS-mediated signaling pathways associated with COX-2.

### 3.7 Anti-inflammatory assay in zebrafish

**FIGURE 9:**
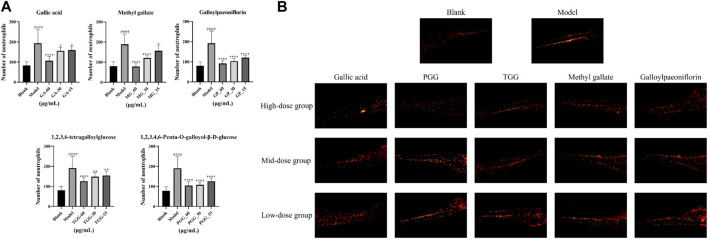
Anti-inflammatory activity of potential inhibitors in zebrafish (*n* = 15, mean ± standard deviation). **(A)** The number of neutrophils in zebrafish was counted under a fluorescence microscope. **(B)** The neutrophils picture of each zebrafish group under the fluorescence microscope. ^####^ *p* < 0.0001, the model group vs. the blank group; ****p < 0.0001, **p < 0.01, *p < 0.05, potential inhibitors group vs. the model group.

In the present study, potential COX-2 inhibitors in MC were preliminarily identified by affinity ultrafiltration and further validated by *in vitro* inhibitory assays. To examine whether the compounds (GA, MG, GP, TGG, PGG) boast *in vivo* anti-inflammatory effect, biological model with LPS-induced inflammation was constructed using transgenic zebrafish. The toxicity of RMC at a series of concentrations was evaluated in 5 days, and a 100% survival rate was attained at 60 μg/mL. Therefore, a gradient consisting of three concentrations (15 μg/mL, 30 μg/mL and 60 μg/mL) was applied for the administration group based on the maximum tolerated concentration. The *in vivo* Anti-inflammatory effect of the compounds were characterized by counting the number of neutrophils in zebrafish. The fluorescence of neutrophils in each group of zebrafish was shown in Figure 9. The results revealed that the number of neutrophils in the model group showed a dramatic rise in comparison to the blank group, indicating that the zebrafish inflammation models were successfully established. Meanwhile, all the experimental groups exhibited a reduction in the number of neutrophils in zebrafish compared with the model group, suggesting that the all the five compounds harbor an *in vivo* anti-inflammatory activity by inhibiting the inflammatory response caused by LPS.

### 3.8 Molecular docking analysis

**FIGURE 10:**
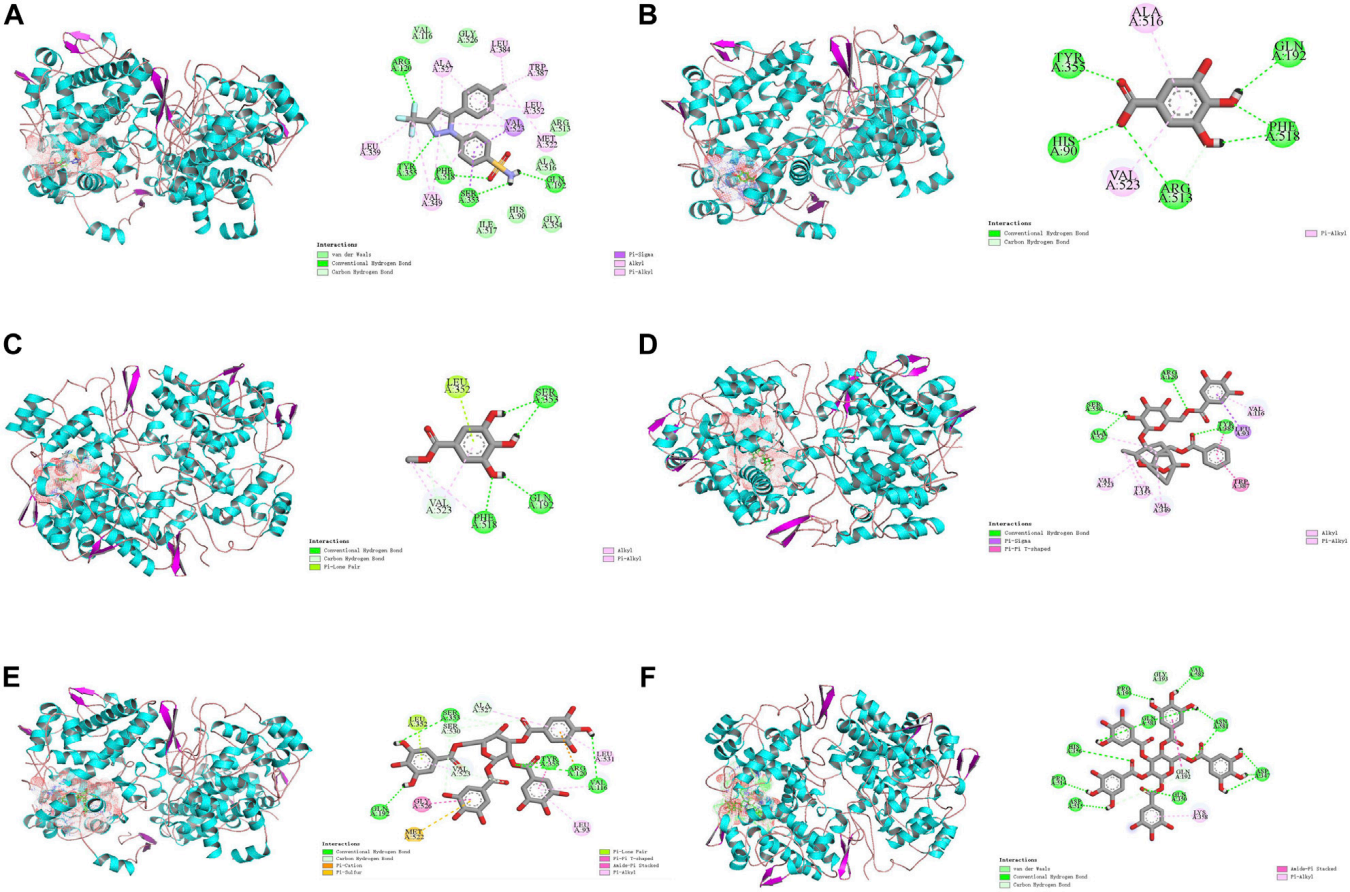
Docking models of screened inhibitors in COX-2 active site. **(A)** celecoxib, **(B)** gallic acid **(C)** methyl gallate, **(D)** galloylpaeonflorin, **(E)** TGG, and **(F)** PGG (Van der Waals are colored in light green; Hydrogen bonds are colored in deep green; Yellow, purple, deep pink, and light pink indicate hydrophobic interactions and represent Pi-Sulful, Pi-sigma, Amide-Pi Stacked, and Pi-Alkyl, respectively).

COX-2 is an active enzyme functioning as cyclooxygenase and peroxidase due to its structure containing the relevant activation sites, each of which is functionally connected by a bridged hemoglobin part (Lucido et al., 2016). According to the literature, when COX-2 produce pro-inflammatory effect, the substrate will form strong hydrogen bonds with Tyr385 and Ser530, as well as van der Waals forces with Arg120. Meanwhile, Trp 387 plays a key role in stabilizing the substrate conformation during the generation of PGG2. Other residues involved in the substrate catalytic process include residues 520-535, Tyr348, Phe381, Tyr385, and Ser530 (Kurumbail et al., 1996; Kiefer et al., 2000). To observe the interaction between the compounds and COX-2, computer simulation-based molecular docking technique was employed in this study. The results of molecular docking in this study were shown in Figure 10, and a summary of the residues interacting with the compound was presented in Table 2. The binding free energy of celecoxib was −9.39 kcal/mol indicating that celecoxib and COX-2 had strong interactions, which indirectly proved that the protein active pocket established in this study was reasonable. The positive drug forms hydrogen bonds with the key residue Arg120 mentioned earlier, while also interacting hydrophobically with Trp387, Tyr355, Val523, Met522, and Ala527. This indicated that celecoxib could enter the active pocket of COX-2, obstructing the entry of substrates and exerting anti-inflammatory effects. Similarly, in the potential anti-inflammatory active compounds in this experiment, GA, MG, GP, and TGG could form hydrogen bonds, van der Waals forces, or hydrophobic interactions with Tyr355, Val523, Ser530, Ala527, Tyr387, and Arg120, respectively. Based on these results, it was speculated that the above compounds, like the positive drug, could enter the active pocket of COX-2 and exerted similar anti-inflammatory effects. While PGG in Figure 10F showed conformations and docking residues indicating that it did not fully enter the interior of the active pocket, the residues with which PGG forms hydrogen bond and van der Waals interactions were mostly located at the entrance of the pocket. This implied that the anti-inflammatory effect produced by PGG was not directly interacting with the key positions in the active pocket, but rather achieving the anti-inflammatory purpose by interacting with residues at the entrance, preventing the substrate from entering the active pocket. Mealwhile, TGG also had lower binding energy with COX-2, but it showed a better *in vitro* COX-2-inhibitory activity in comparison to other inhibitors. The possible explanation was that TGG and PGG, with a larger molecular weight in simulated docking, inevitably had a higher torsional free energy, thus leading to the discrepancy between the results from molecular docking and *in vitro* inhibition assays. Likewise, inconsistent results between the two techniques also occurred in GA, which was associated with the fact that GA was more likely to enter the grid box and interact with the residues during docking due to its smaller molecular weight (Agarwal and Mehrotra, 2016).

**TABLE 2:** Free energy of binding as well as interaction between amino acids and the compounds.

Compound	Estimated free energy of binding (kcal/mol)	Hydrogen bond and pi-pi interaction amino acids	Hydrophobic interaction amino acids
Celecoxib	−9.33	Arg120, Tyr355, Phe518, Ser353, Gln192	Ala527, Leu384, Trp387, Leu352, Val523, Met522, Ser353, Phe518, Val349, Tyr355, Leu359
Gallic acid	−5.46	Tyr355, Arg513, His90, Gln192, Phe518	Val523, Ala516
Methyl gallate	−4.9	Ser353, Gln192, Phe518	Phe518, Val523
Galloylpaeoniflorin	−9.36	Ser530, Ala527, Arg120	Val116, Leu93, Tyr385, Trp387 Val349, Tyr355, Val523
1,2,3,6-Tetra-O-galloyl-β-D-glucose	−4.29	Val116, Gln192, Arg120, Tyr355, Ala527, Ser353, Ser530	Leu93, Leu531, Val523, Leu352, Gly526, Ser530, Met522, Arg120, Tyr355
1,2,3,4,6-Penta-O-galloyl-β-D-glucose	−3.96	Asp515, Pro514, His356, Gln583, Pro191, Val582, Asn581, Asp347, Gln350	Gln192, Lys358

## 4 Conclusion

In this study, an BAUF-UPLC-MS approach on the basis of COX-2 affinity analysis was successfully developed for a rapid, efficient, and targeted screening and identification of the bioactive components in MC. A total of 11 components with potential COX-2-inhibitory activity were discovered. Among them, five compounds—GC, MG, GP, TGG, and PGG—were validated to be bioactive by *in vitro* COX-2-inhibitory experiments. Subsequently, the anti-inflammatory effect of the five components were also assessed with a cell model *in vitro* and zebrafish model *in vivo*. Also, molecular docking was performed to further explore the structure-activity association of the components. Finally, the mechanisms underlying their anti-inflammatory effects were elucidated using network pharmacology approaches. Our results demonstrate that BAUF-UPLC-MS is an effective method for screening active ingredients in complex Chinese medicines. The method is simple, fast, inexpensive, and requires less sample, and can be used for other enzymes to highthroughput screen complex natural products. Also, analyzing these bioactive compounds are useful for quality assessment and clinical application of natural products.

## Data Availability

The original contributions presented in the study are included in the article/Supplementary material, further inquiries can be directed to the corresponding authors.

## Abbreviations

MC, Moutan Cortex; COX-2, cyclooxygenase-2; BAUF-UPLC-MS, bio-affinity ultrafiltration coupled with ultra-performance liquid chromatography-mass; GA, gallic acid; MG, methyl gallate; GP, galloylpaeoniflorin; TGG, 1,2,3,6-Tetra-O-galloyl-β-D-glucose; PGG, 1,2,3,4,6-Penta-O-galloyl-β-D-glucopyranose; DMSO, Dimethyl sulfoxide; TCM, Traditional Chinese Medicine; ESI, electrospray ion; DAD, diode array detector; BD, binding degree; BCA, bicchoninic acid; SDS-PAGE, SDS-polyacrylamide gel electrophoresis; ECL, enhanced chemiluminescent.

## References

[B1] Agarwal S. Mehrotra R. J. J. C. (2016). An overview of molecular docking. JSM Chem. 4 (2), 1024–1028. 10.47739/2334-1831/1024

[B2] Aggarwal B. B. Shishodia S. Sandur S. K. Pandey M. K. Sethi G. (2006). Inflammation and cancer: how hot is the link? Biochem. Pharmacol. 72 (11), 1605–1621. 10.1016/j.bcp.2006.06.029 16889756

[B3] Au T. K. Lam T. L. Ng T. B. Fong W. P. Wan D. C. C. (2001). A comparison of HIV-1 integrase inhibition by aqueous and methanol extracts of Chinese medicinal herbs. Life Sci. 68 (14), 1687–1694. 10.1016/S0024-3205(01)00945-6 11263681

[B4] Bartsch H. Nair J. (2006). Chronic inflammation and oxidative stress in the genesis and perpetuation of cancer: role of lipid peroxidation, DNA damage, and repair. Langenbecks Arch. Surg. 391 (5), 499–510. 10.1007/s00423-006-0073-1 16909291

[B5] Bindu S. Mazumder S. Bandyopadhyay U. (2020). Non-steroidal anti-inflammatory drugs (NSAIDs) and organ damage: a current perspective. Biochem. Pharmacol. 180, 114147. 10.1016/j.bcp.2020.114147 32653589 PMC7347500

[B6] Braun J. Baraliakos X. Westhoff T. (2020). Nonsteroidal anti-inflammatory drugs and cardiovascular risk – a matter of indication. Semin. Arthritis Rheum. 50 (2), 285–288. 10.1016/j.semarthrit.2019.07.012 31439354

[B7] Cai Q. Meng J. Ge Y. Gao Y. Zeng Y. Li H. (2020). Fishing antitumor ingredients by G-quadruplex affinity from herbal extract on a three-phase-laminar-flow microfluidic chip. Talanta 220, 121368. 10.1016/j.talanta.2020.121368 32928397

[B8] Chen S. Dang Y. Gong Z. Letcher R. J. Liu C. (2019). Progression of liver tumor was promoted by tris(1,3-dichloro-2-propyl) phosphate through the induction of inflammatory responses in krasV12 transgenic zebrafish. Environ. Pollut. 255, 113315. 10.1016/j.envpol.2019.113315 31606661

[B9] Chun K. Kim S. O. Lee S. H. (2016). Analgesic effects of 1,2,3,4,6-penta-O-galloyl-β-D-glucose in an animal model of lipopolysaccharide-induced pain. Int. J. Mol. Med. 38 (4), 1264–1270. 10.3892/ijmm.2016.2726 27600119

[B10] Chun S. C. Jee S. Y. Lee S. G. Park S. J. Lee J. R. Kim S. C. (2007). Anti-inflammatory activity of the methanol extract of moutan cortex in LPS-activated Raw264. 7 cells. Evid. Based Complement. Altern. Med. 4 (3), 327–333. 10.1093/ecam/nel093 PMC1978242 17965763

[B11] Ding L. Jiang Z. Liu Y. Chen L. Zhao Q. Yao X. (2012a). Monoterpenoid inhibitors of NO production from Paeonia suffruticosa. Fitoterapia 83 (8), 1598–1603. 10.1016/j.fitote.2012.09.008 23051963

[B12] Ding L. Zhao F. Chen L. Jiang Z. Liu Y. Li Z. (2012b). New monoterpene glycosides from Paeonia suffruticosa Andrews and their inhibition on NO production in LPS-induced RAW 264.7 cells. Bioorg Med. Chem. Lett. 22 (23), 7243–7247. 10.1016/j.bmcl.2012.09.034 23067550

[B13] Federico A. Morgillo F. Tuccillo C. Ciardiello F. Loguercio C. (2007). Chronic inflammation and oxidative stress in human carcinogenesis. Int. J. Cancer. 121 (11), 2381–2386. 10.1002/ijc.23192 17893868

[B14] Fernandes T. A. Antunes A. M. M. Caldeira I. Anjos O. de Freitas V. Fargeton L. (2022). Identification of gallotannins and ellagitannins in aged wine spirits: a new perspective using alternative ageing technology and high-resolution mass spectrometry. Food Chem. 382, 132322. 10.1016/j.foodchem.2022.132322 35158268

[B15] Fitzpatrick F. A. (2001). Inflammation, carcinogenesis and cancer. Int. Immunopharmacol. 1 (9-10), 1651–1667. 10.1016/s1567-5769(01)00102-3 11562058

[B16] He C. N. Peng Y. Zhang Y. C. Xu L. J. Gu J. Xiao P. G. (2010). ChemInform abstract: phytochemical and biological studies of paeoniaceae. Chem. Biodivers. 7 (4), 805–838. 10.1002/cbdv.200800341 20397219

[B17] He Q. Ge Z. W. Song Y. Cheng Y. Y. (2006). Quality evaluation of cortex moutan by high performance liquid chromatography coupled with diode array detector and electrospary ionization tandem mass spectrometry. Chem. Pharm. Bull. 54 (9), 1271–1275. 10.1248/cpb.54.1271 16946533

[B18] Hsiang C. Y. Hsieh C. L. Wu S. L. Lai I. L. Ho T. Y. (2001). Inhibitory effect of anti-pyretic and anti-inflammatory herbs on herpes simplex virus replication. Am. J. Chin. Med. 29 (3-4), 459–467. 10.1142/S0192415X01000472 11789588

[B19] Huai J. Zhao X. Wang S. Xie L. Li Y. Zhang T. (2019). Characterization and screening of cyclooxygenase-2 inhibitors from Zi-shen pill by affinity ultrafiltration-ultra performance liquid chromatography mass spectrometry. J. Ethnopharmacol. 241, 111900. 10.1016/j.jep.2019.111900 31029761

[B20] Jadhav A. S. Shingare M. S. (2005). A new stability–indicating RP-HPLC method to determine assay and known impurity of celecoxib API. Drug Dev. Ind. Pharm. 31 (8), 779–783. 10.1080/03639040500216378 16221612

[B21] Jiao J. J. Yang Y. Z. Wu Z. F. Li B. T. Zheng Q. Wei S. F. (2019). Screening cyclooxygenase-2 inhibitors from Andrographis paniculata to treat inflammation based on bio-affinity ultrafiltration coupled with UPLC-Q-TOF-MS. Fitoterapia 137, 104259. 10.1016/j.fitote.2019.104259 31319108

[B22] Kiefer J. R. Pawlitz J. L. Moreland K. T. Stegeman R. A. Hood W. F. Gierse J. K. (2000). Structural insights into the stereochemistry of the cyclooxygenase reaction. Nature 405 (6782), 97–101. 10.1038/35011103 10811226

[B23] Kurumbail R. G. Stevens A. M. Gierse J. K. McDonald J. J. Stegeman R. A. Pak J. Y. (1996). Structural basis for selective inhibition of cyclooxygenase-2 by anti-inflammatory agents. Nature 384 (6610), 644–648. 10.1038/384644a0 8967954

[B24] Lan Z. Zhang Y. Sun Y. Wang L. Huang Y. Cao H. (2021). Identifying of anti-thrombin active components from curcumae rhizoma by affinity-ultrafiltration coupled with UPLC-Q-exactive Orbitrap/MS. Front. Pharmacol. 12, 769021. 10.3389/fphar.2021.769021 34955839 PMC8703108

[B25] Li J. J. Zhang Y. Han L. W. Tian Q. P. He Q. X. Wang X. M. (2018). Tenacissoside H exerts an anti-inflammatory effect by regulating the nf-κb and p38 pathways in zebrafish. Fish. Shellfish Immunol. 83, 205–212. 10.1016/j.fsi.2018.09.032 30213642

[B26] Liu S. Guo S. Hou Y. Zhang S. Bai L. Ho C. (2023). Chemical fingerprinting and multivariate analysis of Paeonia ostii leaves based on HPLC-DAD and UPLC-ESI-Q/TOF-MS/MS. Microchem. J. 184, 108169. 10.1016/j.microc.2022.108169

[B27] Liu W. Xie G. Yuan G. Xie D. Lian Z. Lin Z. (2021). 6 ’-O-galloylpaeoniflorin attenuates osteoclasto-genesis and relieves ovariectomy-induced osteoporosis by inhibiting reactive oxygen species and MAPKs/c-fos/NFATc1 signaling pathway. Front. Pharmacol. 12, 641277. 10.3389/fphar.2021.641277 33897430 PMC8058459

[B28] Lucido M. J. Orlando B. J. Vecchio A. J. Malkowski M. G. (2016). Crystal structure of aspirin-acetylated human cyclooxygenase-2: insight into the formation of products with reversed stereochemistry. Biochemistry 55 (8), 1226–1238. 10.1021/acs.biochem.5b01378 26859324 PMC4775376

[B29] Matsuda H. Ohta T. Kawaguchi A. Yoshikawa M. (2001). Bioactive constituents of Chinese natural medicines. VI. Moutan cortex. (2): structures and radical scavenging effects of suffruticosides A, B, C, D, and E and galloyl-oxypaeoniflorin. Chem. Pharm. Bull. 49 (1), 69–72. 10.1248/cpb.49.69 11201228

[B30] Mohan M. Hussain M. A. Khan F. A. Anindya R. (2021). Symmetrical and un-symmetrical curcumin analogues as selective COX-1 and COX-2 inhibitor. Eur. J. Pharm. Sci. 160, 105743. 10.1016/j.ejps.2021.105743 33540041

[B31] Ow Y. Y. Stupans I. (2003). Gallic acid and gallic acid derivatives: effects on drug metabolizing enzymes. Curr. Drug Metab. 4 (3), 241–248. 10.2174/1389200033489479 12769668

[B32] Qin S. Ren Y. Fu X. Shen J. Chen X. Wang Q. (2015). Multiple ligand detection and affinity measurement by ultrafiltration and mass spectrometry analysis applied to fragment mixture screening. Anal. Chim. Acta 886, 98–106. 10.1016/j.aca.2015.06.017 26320641

[B33] Sheehan M. P. Atherton D. J. (1994). One-year follow up of children treated with Chinese medicinal herbs for atopic eczema. Brit J. Dermatol 130 (4), 488–493. 10.1111/j.1365-2133.1994.tb03383.x 8186115

[B34] Smalley W. E. DuBois R. N. (1997). Colorectal cancer and nonsteroidal anti-inflammatory drugs. Adv. Pharmacol. 39, 1–20. 10.1016/s1054-3589(08)60067-8 9160111

[B35] Strippoli V. D’Auria F. D. Tecca M. Callari A. Simonetti G. (2000). Propyl gallate increases *in vitro* antifungal imidazole activity against Candida albicans. Int. J. Antimicrob. Ag. 16 (1), 73–76. 10.1016/S0924-8579(00)00200-4 11185418

[B36] Watanabe T. Fujiwara Y. Chan F. K. L. (2020). Current knowledge on non-steroidal anti-inflammatory drug-induced small-bowel damage: a comprehensive review. J. Gastroenterol. 55 (5), 481–495. 10.1007/s00535-019-01657-8 31865463 PMC7188723

[B37] Wei H. Zhang X. Tian X. Wu G. (2016). Pharmaceutical applications of affinity-ultrafiltration mass spectrometry: recent advances and future prospects. J. Pharm. Biomed. 131, 444–453. 10.1016/j.jpba.2016.09.021 27668554

[B38] Wolfender J. L. Marti G. Thomas A. Bertrand S. (2015). Current approaches and challenges for the metabolite profiling of complex natural extracts. J. Chromatogr. A 1382, 136–164. 10.1016/j.chroma.2014.10.091 25464997

[B39] Xiong P. Qin S. Li K. Liu M. Zhu L. Peng J. (2021). Identification of the tannins in traditional Chinese medicine paeoniae radix alba by UHPLC-Q-exactive Orbitrap MS. Arab. J. Chem. 14, 103398. 10.1016/j.arabjc.2021.103398

[B40] Xu H. Zhang X. Li H. Li C. Huo X. J. Hou L. P. (2018). Immune response induced by major environmental pollutants through altering neutrophils in zebrafish larvae. Aquat. Toxicol. 201, 99–108. 10.1016/j.aquatox.2018.06.002 29902668

[B41] Yun C. S. Choi Y. G. Jeong M. Y. Lee J. H. Lim S. (2013). Moutan Cortex Radicis inhibits inflammatory changes of gene expression in lipopolysaccharide-stimulated gingival fibroblasts. J. Nat. Med. 67 (3), 576–589. 10.1007/s11418-012-0714-3 23086154

[B42] Zhang X. Wei J. Que Z. Chen Y. (2017). Research progress on anti-inflammatory effects of traditional Chinese medicine. Chin. J. Ethnomedicine Ethnopharmacy 26 (21), 67–69.

[B43] Zhang X. Y. Wei Z. J. Qiao W. L. Sun X. M. Jin Z. L. Gong W. (2021). Discovery of cyclooxygenase-2 inhibitors from Kadsura coccinea by affinity ultrafiltration mass spectrometry and the anti-inflammatory activity. Fitoterapia 151, 104872. 10.1016/j.fitote.2021.104872 33657428

